# Knockout of longevity gene *Sirt1* in zebrafish leads to oxidative injury, chronic inflammation, and reduced life span

**DOI:** 10.1371/journal.pone.0220581

**Published:** 2019-08-06

**Authors:** Do Hee Kim, In Hye Jung, Dong Hee Kim, Seung Woo Park

**Affiliations:** 1 Postgraduate School of Nano Science and Technology, Yonsei University, Seoul, Korea; 2 Department of Internal Medicine, Yonsei University College of Medicine, Institute of Gastroenterology, Severance Hospital, Seoul, Korea; National Institutes of Health, UNITED STATES

## Abstract

*Sirt1*, a member of the sirtuin gene family, encodes the most conserved mammalian NAD^+^-dependent deacetylase enzyme responsible for removing acetyl groups from many proteins. The *Sirt1* gene is known as a longevity gene whose knockout in mice leads to decreased lifespan relative to the wild type. This study aimed to explore phenotypic changes in zebrafish *Sirt1*-knockouts and to investigate the function of the Sirt1 gene. Targeted knockout of *Sirt1* in zebrafish (*Danio rerio*) was achieved using the CRISPR-Cas9 genome editing system. We created a 4-bp insertion-homozygote *Sirt1*-knockout zebrafish. Although there was no evident difference in appearance in the early stages of development, a significant increase in reactive oxygen species and in the extent of apoptosis in *Sirt1*-knockout zebrafish was observed. Sirt1 knockout caused inflammatory genes, including IL-1b, IL-6 and TNF-α to be highly expressed. Additionally, the lack of Sirt1 caused chronic inflammation and intestinal atrophy, thereby increasing pro-apoptotic events, which ultimately reduced the lifespan of transgenic zebrafish. Overall, our data demonstrate that lack of *Sirt1* caused a significantly increased generation of reactive oxygen species that resulted in chronic inflammation and regeneration. Continuous repetition of these events played an important role in accelerating aging, thereby decreasing lifespan. Our findings using the knockout zebrafish model confirmed the association of the *Sirt1* gene to aging processes and lifespan. Furthermore, the *Sirt1*-knockout mutant zebrafish developed in our study will surely be a valuable model to explore the mechanism of chronic inflammation.

## Introduction

Aging is related to chronic inflammation and generation of reactive oxygen species (ROS). Sirtuin 1 (*Sirt1*), the longevity gene, is a member of the sirtuin family [1] encoding a nicotinamide adenine dinucleotide (NAD)-dependent classⅢ histone deacetylase that removes acetyl groups from histone and non-histone proteins. *Sirt1* is induced under metabolically stressful environments that are deficient in nutrients or oxygen. Members of the SIR proteins family (sirtuins) are NAD^+^-dependent protein deacetylases or ADP-ribose transferases that have evolved extensively in most biological systems, including plants, bacteria, and animals. SIR2 was first discovered in yeast-mating studies and was reported to be involved in a wide array of cellular processes, including telomeric and ribosomal DNA (rDNA) silencing, [2–4] intracellular signaling regarding cell cycle and senescence, and in the regulation of metabolism by dephosphorylating not only histones, but various transcription factors and cofactors as well [5,6]. Furthermore, SIR2 functions in longevity, muscle differentiation and DNA damage repair. Previous lifespan studies showed that overexpression of *Sir2* induced an extension of the lifespan in yeast, drosophila and mice [7–9]; *Sirt1*—a mammalian ortholog of *Sir2*—knockout mice exhibited developmental abnormalities in several organs and a significantly shorter lifespan, compared with wildtype mice [10]. The action of the *Sirt1* gene against aging-related disease promotes an extension of the lifespan and presumably occurs by an increase in stress resistance and gene expression-mediated protection from cell death.

As most studies on *Sirt1* have associated lifespan with *Sirt1* knockout in transgenic mice under conditions of caloric restriction [4,11], it is difficult to explain what role the *Sirt1* gene might play in longevity. Therefore, in this study, we generated *Sirt1*-knockout zebrafish using CRISPR/Cas9 and explored the phenotypic changes in these knockouts, relative to the wildtype, in an attempt to elucidate the function of the *Sirt1* gene.

## Materials and methods

### Knock-out model construction using CRISPR-cas9

We used pT7gRNA (Addgene) and pRGEN-Cas9-CMV (Toolgen) to generate sgRNA and Cas9 mRNA, respectively. CRISPR/Cas9 target site design was done using online site E-crisp(www.e-crisp.org) and Exon1 of the zebrafish *Sirt1* target. The selected nucleotides were cloned into pT7gRNA (Addgene) using primers Sirt1-F(5’-TAGGACGAGAAACCGGCGCGGA- 3’), Sirt1-R(5’-AAACTGCTCTTTGGCCGCGCCT- 3’). RNA was linearized with *BamHI* and *in vitro* transcription was carried out using the MEGAshortscript T7 kit (Ambion. Inc., Austin, TX). For microinjection, 50 ng/μl of Cas9 RNA, 150 ng/μl of gRNA, 20 mM Hepes, and 150 mM KCl containing 0.03% phenol red were prepared and then introduced into one-cell AB strain zebrafish embryos. The F1 progenies were genotyped by sequence analysis of the genomic DNA isolated by tail fin clipping to screen for the germ line mutation. Primer sequences used to amplify the exon1 sequences were 5’-CGAAAATAAACGGGCCGAA- 3’ (forward), and 5’-AGATCTCGGGCTCCGGGTC- 3’ (reverse). The identified heterozygote mutant zebrafish were self-crossed to produce F2 progenies. The homozygote *Sirt1*-null zebrafish were screened from F3 progenies produced by inbreeding of the F2 heterozygote progenies.

### Animal stocks and embryo care

Wildtype (AB strain) and *Sirt1*^*-/-*^ zebrafish were raised in a standardized aquaria system (Genomic-Design Co., Daejeon, Korea) (http://zebrafish.co.kr). The system provided continuous water flow, biofiltration tank, at a constant temperature of 28.5°C, UV sterilization, and 14/10 h light/dark cycle. Embryos to be processed for whole-mount analyses were placed in an E3 medium with 0.003% phenylthiourea at 24 h after fertilization to inhibit pigmentation. Embryos and adult zebrafish were anaesthetized in 0.02% tricaine (3-aminobenzoic acid ethyl ester, Sigma) before sacrifice for analysis. This study was approved by Institutional Animal Care and Use Committee of Yonsei University Health System (IACUC of YUSH: 2019–0036).

### Drug treatment

Four-days post fertilization (dpf) zebrafish embryos were separated into different treatment groups. Fifteen embryos of each group were incubated in E3 embryo medium. Except for the control group, the other treatment groups were exposed to tBOOH (0.5–1 mM) and N-acetylcysteine (NAC, at 1 μM). Fish survival was monitored for 6 days at 6–12 h intervals, for the ROS sensitivity test.

### ROS detection and TUNEL assay

The cell-permeant 2´7´-dichlorodihydrofluorescein diacetate H_2_DCFDA (Invitrogen, OR, USA) was used to detect ROS in live embryos. Four-dpf drug-treated or untreated embryos were incubated with 10 μM H_2_DCFDA for 20 min at 0.5 h prior to confocal imaging. Embryos were anaesthetized for live imaging. For analysis of cell apoptosis, the *in situ* cell-death detection kit, TMR red (Roche Diagnostics GmnH) was used. Briefly, 4% paraformaldehyde fixation was done with 0.02% buffered Tricaine in egg water. Paraformaldehyde-fixed embryos were washed with PBS three times and then treated with 0.1% Collagenase P (Roche, Germany) for 1 h at room temperature; PBS wash was repeated, and embryos were labeled with TUNEL reaction mixture overnight at 4°C. After washing three times with PBS, embryos were analyzed using a Zeiss LSM700 and LSM710 laser-scanning confocal microscope. All data were obtained from at least three independent experiments.

### Real-time PCR and Western blot analysis

Real-time PCR was performed using whole zebrafish embryos and 3-month-old dissected zebrafish liver and intestine. RNA samples were extracted using TRIzol reagent (Invitrogen, Fisher Scientific, CA); cDNA was synthesized from 2 μg of total RNA with a Maxima First Stand cDNA Synthesis Kit (Thermo Fisher Scientific, K1641, Glen Burnic, MD); real-time PCR was performed using Maxima SYBR Green/ROX qPCR Master Mix (Thermo Fisher Scientific, K0222) on a 7300 Real-Time PCR System (Applied Biosystems, Foster city, CA). The primer sequences for Real-time PCR are shown in Table 1.

**Table 1:** qPCR primers.

Gene	Forward	Reverse
β-actin	ATGGATGAGGAAATCGCTGC	CTTTCTGTCCCATGCCAACC
IL-1β	CATGCGGGCAATATGAAGTC	CATTTTGTGCTGCGAAGTCC
IL-6	ATGCCATCCGCTCAGAAAACAG	CCAAGGAGACTCTTTACGTCCA
IL-8	ATGACCAGCAAAATCATTTCAGTGTG	AAATCTTTACAGTGTGGGCTTG
IFN-γ	ATGGATTCCTGCCTCAAAATG	TCCAACCCAATCCTTTGCAA
TNF-α	ATGAAGCTTGAGAGTCGGGC	TGTTGATTGCCCTGGGTCTT
TGF-β1a	GTTGGTTTGCTTGGTGCTGA	ATCTTCTGTCCGTCGTCGTC
TGF-β2	TGAACTTGTACGTCTTGAGC	GATCTCAGGAGGACTGCTCA
NOS2	ATGGGAAGACAAGCACAAACCA	GCCGCTTTGTGATGAAGTGA
MMP2	GTTGAAGGACACGCTGAAGAAA	GGGTGTGCCCTAAGATTCTG
MMP9	ATGAGACTTGGAGTCCTGGC	TTAGCATTGGAGATGACCGC
TIMP2	TGAAGAGCGTCAGGAGCTGTA	GCTTGATCGGGTTCCCATAA
SOCS1a	ATGGTGGCGCACAGTTCAGT	TGCATAGTTGAACGGCTTGA
SOCS3a	TGATAACCCACAGCAAGTTG	AGCAGGTGGTTGGCCTCTTT
Nfe2L2	ATGATGGAGATTGAAATGTC	AGTGTCTTCTCCTGCTCCTG
SOD1	ATGGTGAACAAGGCCGTTTG	AGGGTTGAAGTGCGGACCTG
Sirt1	ATGGCGGACGGCGAAAATAA	CGGTCTAGTGTTGTTGTTGTTG
PGC1a	ATGGCGTGGGACAGGTGTAA	CGCTGTACCACTTGAGTCCT
PTGS2a	TGAATAAACTGGTTTGCTTGGTCC	TTGATGCGTGTGAGAAGCTCA

For western blot analysis, whole cell extracts were prepared from zebrafish embryos (N = 50 per group) and lysed with lysis buffer (50 mM Tris-HCL (pH 7.9), 100 mM NaCl, 1 mM EDTA, 2% SDS, 0.1 mM EDTA, 0.1 mM EGTA, and 0.1 M protease and phosphatase inhibitor cocktail (Thermo Scientific); 20-μg total protein samples were treated with Laemmli sample buffer, heated at 100°C for 5 minutes, and loaded into each well on the stacking gel for separation by 8% and 12% SDS-PAGE and electroblotted onto nitrocellulose membranes. Membranes were blocked in 5% non-fat dry milk in PBS-T and incubated with primary antibodies overnight at 4°C. They were then probed with peroxidase-conjugated secondary antibody for 1 h at room temperature. The primary antibodies included rabbit polyclonal anti-Sirt1 (Aviva systems Biology Co, San Diego, CA, USA, 1:1000), rabbit polyclonal anti-IL-1b (Abcam, Cambridge, MA, USA, 1:1000), rabbit polyclonal anti-caspase3a (Anaspec, Fremont, CA, USA, 1:1000), rabbit polyclonal anti-TGF-β (Anaspec, 1:1000) and rabbit monoclonal anti-β-actin (EPR6255, Abcam, 1:2000). The secondary antibody included goat anti-rabbit IgG (H+L)-HRP (GenDepot, Katy, TX, USA,1:5000). The washes were repeated, and the membrane developed with a chemiluminescent agent (ECL; Amersham Life Science, Inc.). Band density was measured using TINA image software (Raytest, Straubenhardt, Germany). Real-time PCR and western blot analysis were performed at least three times using separately prepared samples.

### Histology, immunohistochemistry (IHC), and *in situ* hybridization

After anaesthetizing 6-, 12-, and 18-month-old zebrafish (N = 8 per each stage of group) were sacrificed for histology. Histological evaluation was done using 4-μm sections of paraformaldehyde-fixed and paraffin-embedded tissue. Hematoxylin and eosin (H&E) staining and IHC were performed according to standard protocols. IHC and ISH experiments were carried out as previously described [12,13]. For IHC experiments, rabbit polyclonal anti-myeloperoxidase (Abcam, 1:1000) was used.

Riboprobes were generated by PCR amplification with partial cDNA (707-bp), sequences of F-zp65 (5’-TGGACGGAATGTTTCACCAG- 3’), R-zp65 (5’-CTAATACGACTCACTATAGGGCTCTCCCACGAGTCCAGGAA-3’), F-zTNF-α (5’-ATGAAGCTTGAGAGTCGGGC-3’), and R-zTNF-α (5’-CTAATACGACTCACTATAGGGCGTGTCTGTGCCCAGTCTGT- 3’), *in vitro* transcription was carried out using mMESSAGE mMACHINE T7 ultra Kit (Ambion, Inc., Austin, TX). Hybridization was done at 60°C overnight, and serial stringent washing was done at 68°C; the hybridized riboprobe was detected by anti-dig antibody binding and detected by NBT/BCIP AP substrate solution (Roche Diagnostics GmnH). Finally, counterstaining was done with neutral red [12,14].

### Kaplan-Meier analysis

Wildtype and *Sirt1*^-/-^ zebrafish were monitored for survival analysis (N = 100 per group). The observed survival duration ranged from 3 to 18 months and was considered as 50%. Zebrafish were regularly checked.

### Statistical analysis

Data are presented as means ± standard deviation (SD) from at least three independent experiments. Significant differences among groups were determined by Student’s t-test. Values at *P < 0.05 were considered statistically significant.

## Results

### Generation of *Sirt1*-knockout zebrafish

**Fig 1:**
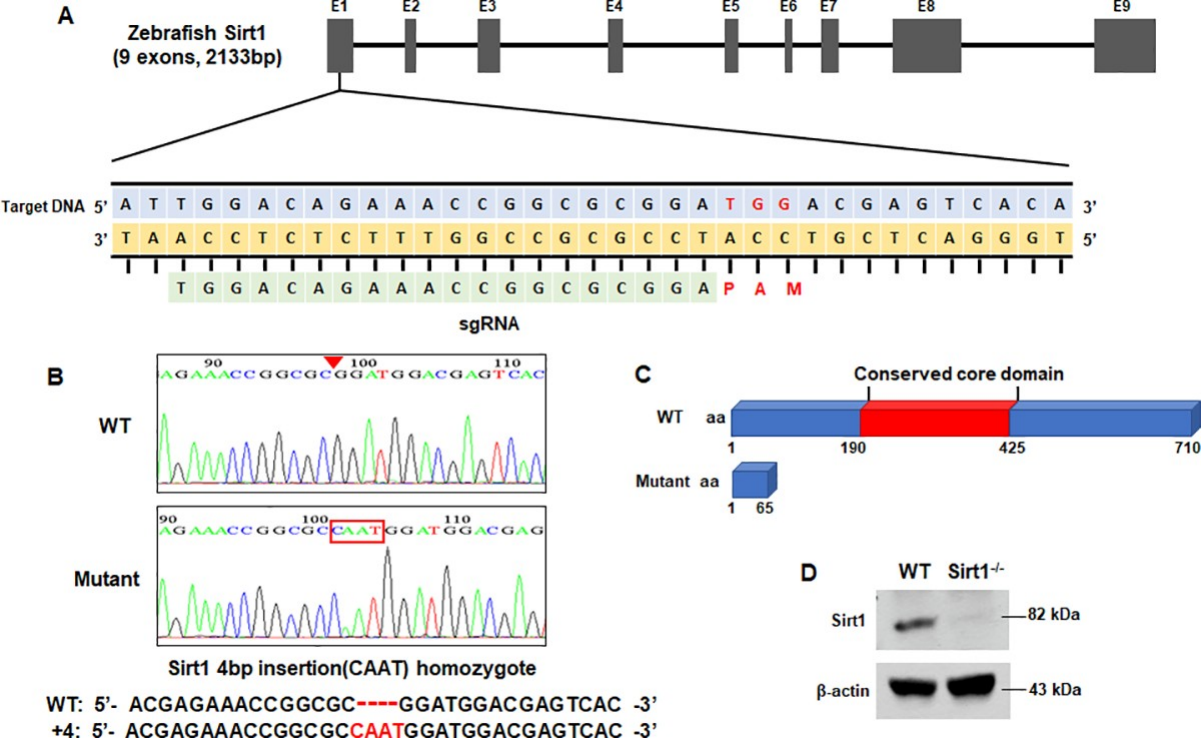
*Sirt1* Knockout by Crispr/Cas9 technique. (A) Schematic illustration of the *Sirt1* locus. Exon 1 of the *Sirt1* gene was targeted. The CRISPR target site and PAM motif are indicated. (B) Sequence confirmation of four-nucleotide insertion mutations. (C) Predicted truncation of the SIRT1 protein. (D) Expression of the *Sirt1* mutant was verified by western blot using an antibody of *Sirt1*; β-actin was used as a loading control.

The CRISPR/Cas9 system was used to generate *Sirt1*-knockout zebrafish. We designed and generated a sgRNA against exon1 of the *Sirt1* zebrafish gene (Fig 1A). The sgRNA and the Cas RNA were injected into the yolk of AB zebrafish at the one-cell stage of development; F1 fish were generated by backcrossing the F0 adult zebrafish. We performed the genotype and found the insertion of 4-bp (CAAT) in F1 heterozygotes. The identified heterozygote mutant zebrafish were backcrossed again with AB zebrafish to produce F2 progenies. The homozygote *Sirt1*-knockout zebrafish were screened out from the F3 progenies produced by inbreeding of the F2 heterozygote progenies. DNA sequencing results confirmed the targeted four-nucleotide insertion mutations (Fig 1B). These mutations led to a truncation of the *Sirt1* protein, resulting in a protein that lacked a catalytic domain (Fig 1C). We confirmed the expression of the *Sirt1* protein by western blot. For western blot analysis, 4-dpf embryos were used for total protein extraction. The SIRT1 protein was completely absent in the *Sirt1*^*-/-*^ mutant zebrafish embryos (Fig 1D). Thus, our analysis demonstrated that homozygous *Sirt1*-knockout zebrafish were successfully generated.

### *Sirt1* knockout induced ROS and apoptosis

**Fig 2:**
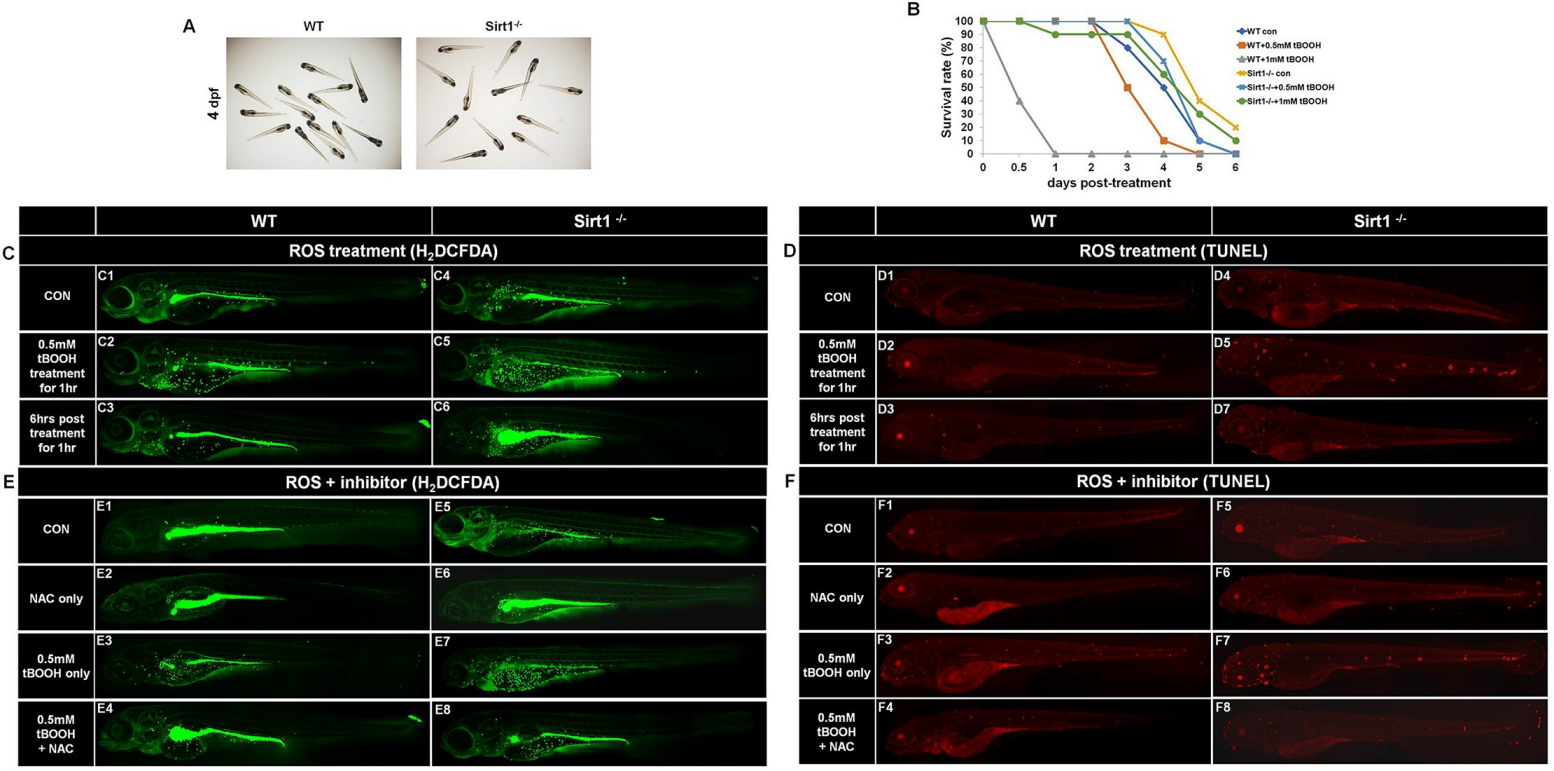
Short term phenotype of *Sirt1* ^-/-^ mutants. Four-days post fertilization embryos were used to obtain representative images of short term phenotypes of wildtype and *Sirt1*^-/-^ mutants. (A) Phenotypes of wildtype and *Sirt1*^-/-^ zebrafish embryos. Note there are no differences in appearance between wildtype and *Sirt1*^-/-^ mutant embryos. (B) The embryo survival curves of wildtype and *Sirt1*^-/-^ mutants after treatment with 0.5 or 1 mM tBOOH. Note that wildtype embryos died earlier than *Sirt1*^-/-^ mutants. (C) ROS levels in 0.5 mM tBOOH-treated wildtype and *Sirt1*^-/-^ embryos were examined by H_2_DCFDA fluorescence. (D) Detection of apoptosis in 0.5 mM tBOOH-treated wildtype and *Sirt1*^-/-^ mutant embryos by TdT-mediated dUTP nick end labeling (TUNEL). Note that *Sirt1*^-/-^ mutants showed higher levels of ROS and TUNEL than wildtype embryos. The effect of NAC treatment on ROS content and apoptosis incidence was studied by H_2_DCFDA fluorescence (E) and the TUNEL assay (F). Embryos were exposed to treatment with NAC treatment for 24 h. Results showed that TUNEL-positive cells increased in both, tBOOH-treated wildtype and *Sirt1*^-/-^ groups; furthermore, NAC treatment was effective for both groups.

Wildtype and *Sirt1*^-/-^ embryo phenotypes were assessed at 4 dpf. Compared with wildtype embryos, *Sirt1*^-/-^ showed no apparent phenotypic difference. (Fig 2A). Among known ROS effects, increased ROS levels can directly or indirectly control the activity of the SIRT1 enzyme [15]. To identify ROS sensitivity, we treated 4-dpf zebrafish embryos with *tert*-butyl-hydroperoxide (tBOOH) as an oxidant to produce oxidative damage stress, and then we recorded the number of surviving zebrafish (Fig 2B). We expected tBOOH-treated *Sirt1*^-/-^ embryos to die earlier than wildtype embryos. However, the biological outcome was different: one day after treatment with 1 mM tBOOH, all wildtype embryos were dead, whereas *Sirt1*^-/-^ embryos showed 90% survival rate. Consistently, only 50% of wildtype embryos survived after 3 days of treatment with 0.5 mM tBOOH, whereas 80% of *Sirt1*^-/-^ embryos survived under the same treatment.

We then examined ROS production in wildtype and *Sirt1*^-/-^ embryos after tBOOH treatment (Fig 2C). ROS production was detected in 4-dpf embryos by H_2_DCFDA fluorescence. We divided embryos in three sets and performed parallel treatments. One set received no treatment (control), a second set was treated with 0.5 mM tBOOH for 1 h, whereas the third set was treated with 0.5 mM tBOOH for 1 h and observed 6 h after the media was changed. Compared with wildtype embryos, ROS level in *Sirt1*^-/-^ mutant embryos was higher than that in the control group (Fig 2C1 and 2C4). After 0.5 mM tBOOH treatment for 1 h, ROS levels in both wildtype and *Sirt1*^-/-^ embryos had increased over the level observed in each control group, but the 0.5 mM tBOOH-treated *Sirt1*^-/-^ group showed the highest ROS content (Fig 2C2 and 2C5). However, at 6 h post 0.5 mM tBOOH treatment for 1 h, ROS content had decreased in both; wildtype and *Sirt1*^-/-^ mutant zebrafish embryos (Fig 2C3 and 2C6).

We next examined cell death after tBOOH treatment using TUNEL (Fig 2D). This showed a significant mean effect of the lack of *Sirt1* in the control group (Fig 2D1 and 2D4). There was a significant induction of TUNEL-positive cells in tBOOH-treated wildtype and *Sirt1*^-/-^ embryos (Fig 2D2 and 2D5). At 6 h post 0.5 mM tBOOH-treatment for 1 h, wildtype and Sirt1^-/-^ embryos recovered from cell death (Fig 2D3 and 2D6).

For confirmation of the relationship between ROS and apoptosis in Sirt1^-/-^ mutants, the surviving 4-dpf zebrafish embryos were subjected to H_2_DCFDA fluorescence and TUNEL assay after treatment with NAC, a commonly used ROS inhibitor [16], and the images were observed under confocal microscope. Fig 2E and 2F shows the effect of NAC (1 μM) treatment on zebrafish embryos for 24 h.

In the wildtype group, no variation between NAC-only treatment and the control was observed regarding ROS content and apoptosis (Fig 2E1 and 2E2 and Fig 2F1 and 2F2). In contrast, tBOOH + NAC treatment reduced ROS content and cell death compared to tBOOH-only treatment (Fig 2E3 and 2E4 and Fig 2F3 and 2F4). Furthermore, in the *Sirt1*^-/-^ group, NAC abated ROS content and cell death rate induced by the lack of *Sirt1* (Fig 2E5 and 2E6 and Fig 2F5 and 2F6). Co-administration of tBOOH and NAC significantly reduced tBOOH-induced oxidative injury (Fig 2E7 and 2E8 and Fig 2F7 and 2F8). These findings clearly demonstrated that our *Sirt1*^-/-^ mutant zebrafish model can be used for inflammatory drug testing.

### Lack of *Sirt1* caused upregulation of inflammation-involved genes in the early stages of embryo development

**Fig 3:**
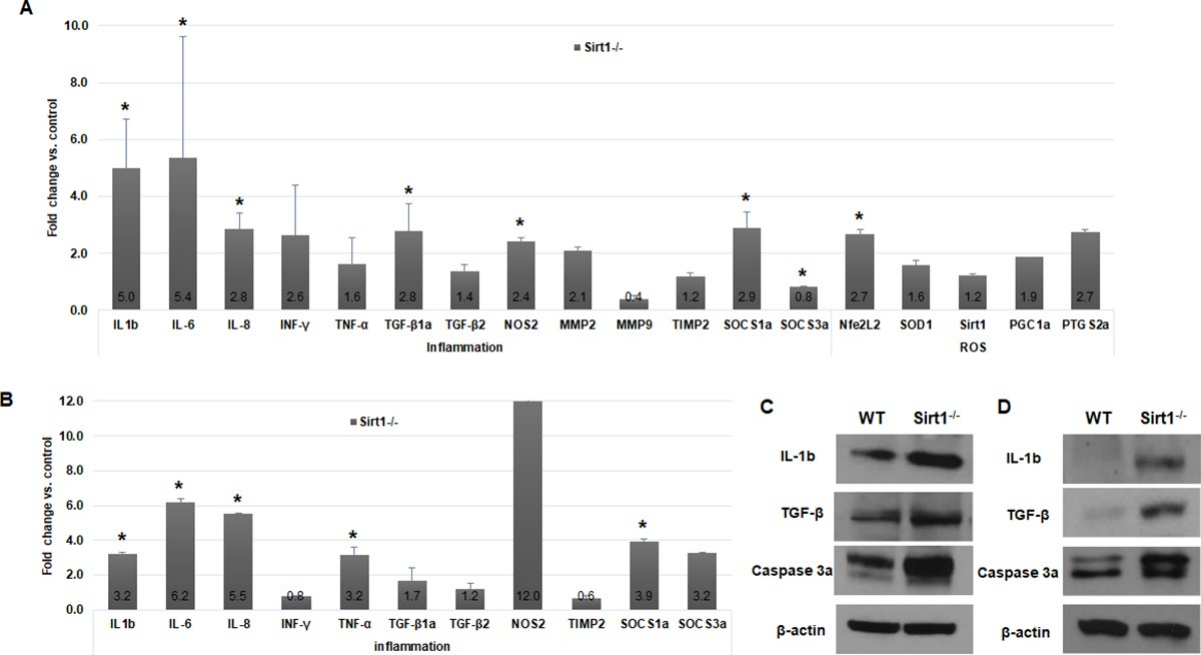
Quantitative RT-PCR and western blot analysis. Samples were prepared from (A, C) 4-dpf whole embryos, and (B, D) internal organs from 3-month-old zebrafish. Data are means, and bars indicate standard error. (A, B) Quantitative RT-PCR showed higher upregulation of inflammation-related genes in *Sirt1*^-/-^ than in controls. (C, D) Western blot hybridization showed upregulation of IL-1b, active-caspase 3 and TGF-β. Control, wildtype; IL-1b, 18kDa; caspase 3a, 29 and 31 kDa; TGF-β, 45 kDa; β–actin, 43 kDa. *P < 0.05 versus control.

To identify differentially expressed genes induced by the lack of *Sirt1*, 4-dpf whole embryos (Fig 3A) and dissected internal organs from 3-month-old zebrafish (Fig 3B) were processed for quantitative RT-PCR. We selected a list of genes involved in inflammation and ROS. (Primers are listed in Table 1). As seen in Fig 3A and Fig 3B, quantitative RT-PCR revealed higher upregulation of most inflammation-related genes, including IL-1b, IL-6, IL-8, TNF-α, and SOCS1a, in *Sirt1*^-/-^ mutants, compared with that in the wildtype. Western blot hybridization carried out using samples from 4-dpf whole embryos (Fig 3C) and 3-month-old zebrafish (Fig 3D) with antibodies reactive to zebrafish antigen confirmed the RT-PCR results. These results indicated that the loss of *Sirt1* induced inflammation gene expression from the early stages of embryo development.

### Effects of the lack of *Sirt1* in old aged adult zebrafish

**Fig 4:**
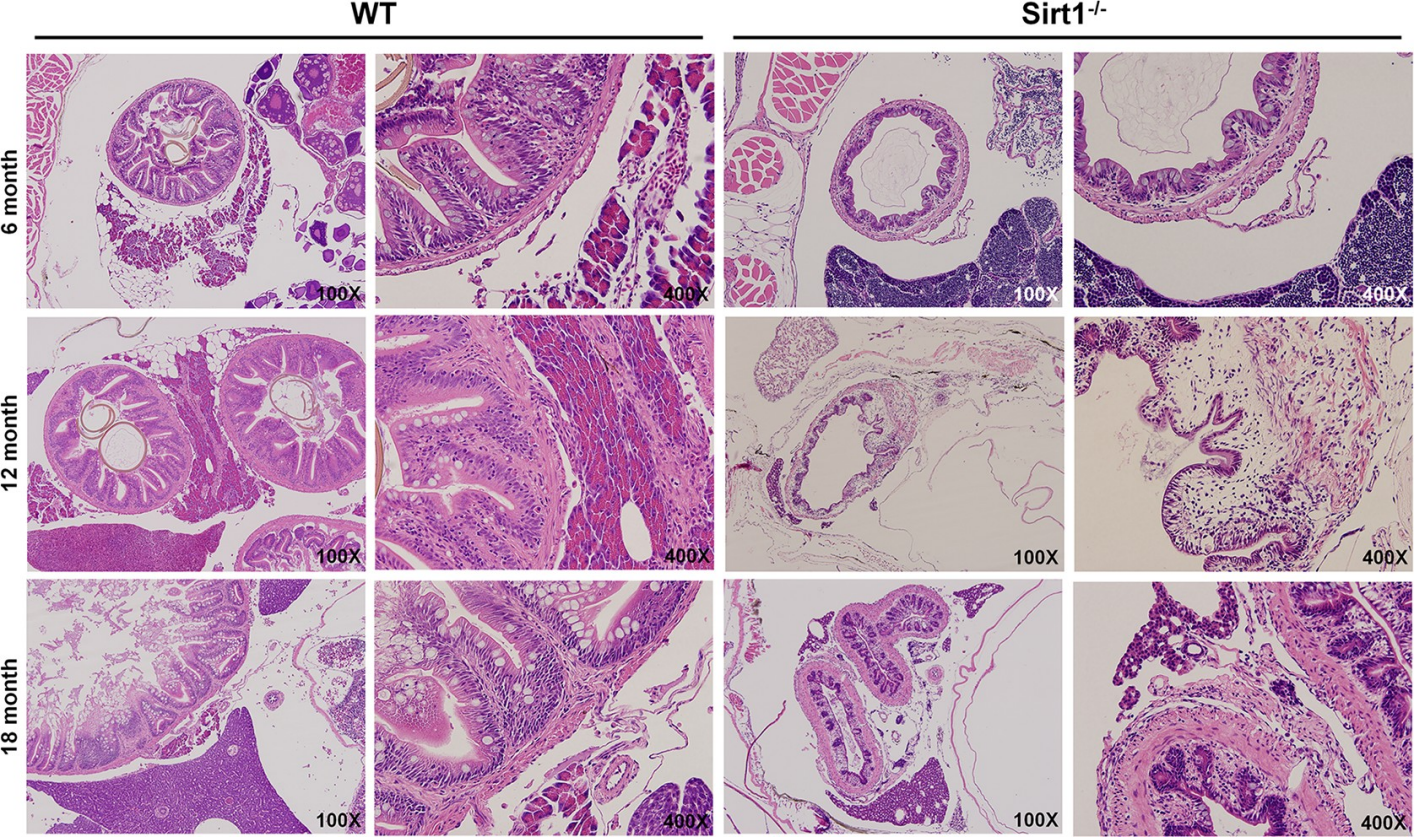
Microscopic observations of wildtype and *Sirt1*^-/-^ adult zebrafish. H&E images of wildtype and *Sirt1*^-/-^ zebrafish aged 6, 12, and 18 months. *Sirt1*-deficiency caused intestinal atrophy and inflammatory cell infiltration.

In order to characterize any other phenotypic changes indicated by histological analysis we conducted microscopic observations on intestine sections from 6-month-old *Sirt1*^-/-^ zebrafish (Fig 4). We found that inflammatory cell infiltration occurred in the adjacent organs including the pancreas, intestines, and spleen. Additionally, all *Sirt1*^-/-^ zebrafish revealed a varying degree of intestinal atrophy. To further verify whether *Sirt1* deficiency contributed to inflammation, we examined inflammatory expression in zebrafish sections.

Histological expression of inflammation was assessed by either IHC or ISH on 12-month old zebrafish sections. Firstly, using an antibody against Myeloperoxidase (MPO), a well know enzyme characterized by pro-oxidative and proinflammatory properties [17], we confirmed a higher level of MPO expression in *Sirt1*^-/-^. Next, ISH staining against NF-kB and TNF-α were examined to address any potential involvement of these inflammatory mediators. Altogether, these results supported the finding that loss of *Sirt1* in zebrafish ultimately led to inflammation.

### *Sirt1*^-/-^ survival assessment by the Kaplan-Meier method

**Fig 5:**
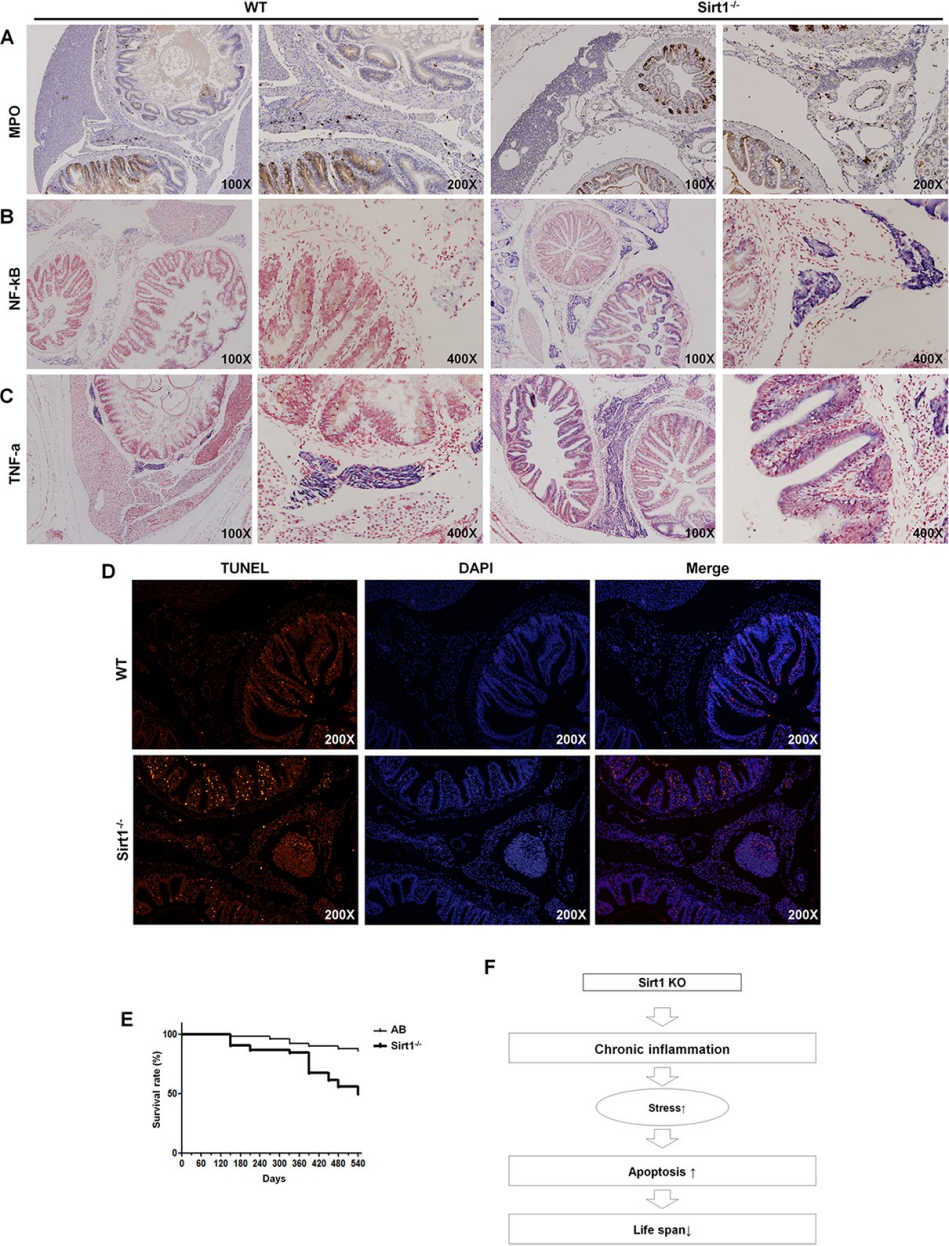
Expression of genes involved in inflammation and apoptosis induced by *Sirt1*-knockout induced inflammation. All images were obtained from 12-month-old zebrafish. (A) Immunohistochemical staining for myeloperoxidase (MPO). (B) ISH for NF-kB and (C) TNF-α expression. (D) TUNEL assay with 18-month old zebrafish organ sections. Wildtype organ section with few apoptotic cells and *Sirt1*^-/-^ organ section with numerous apoptotic cells, as per the TUNEL assay. (E) Survival curves of wildtype and *Sirt1*^-/-^ mutant zebrafish. Kaplan–Meier analysis showed poor survival of *Sirt1*^-/-^ compared to the wildtype. (n = 100 zebrafish in each group). Survival curves lasted for 540 days (observation started when zebrafish were 3 months old.) and were statistically compared using the Log-Rank Test. (F) Schematic diagram of the major findings of this study. *Sirt1*^-/—^induced chronic inflammation led to the activation of oxidative stress and apoptosis. The continuous repetition of these events reduced the lifespan of zebrafish.

The *Sirt1* gene is well-known to correlate with longevity [9,18]. Aging is characterized by a systemic, chronic, pro-inflammatory condition with rising levels of TNF-α, IL-1, and IL-6 [19]. We therefore verified whether our *Sirt1*^-/-^ mutants showed a strong correlation with aging; thus, wildtype and *Sirt1*^-/-^ zebrafish were used for survival analysis. The observed survival duration ranged from 3 to 18 months and was considered as 50%. We found that survival of *Sirt1*^-/-^ mutant zebrafish was similar to that of the wildtype up to 5 months of age; then it slightly decreased until 7 months of age, and was greatly reduced among 13-month-old fish. The death of *Sirt1*^-/-^ mutant zebrafish peaked roughly at 16 months of age. Overall, the survival rate of *Sirt1*^-/-^ mutant zebrafish was significantly lower than that of the wildtype (Fig 5E). Concomitantly, apoptotic events were confirmed to occur using the TUNEL assay on 18-month-old zebrafish organic sections. We recorded a higher number of apoptotic cells occurring in *Sirt1*^-/-^ mutants than in the wildtype (Fig 5D). Altogether, these results suggest that the observed reduction in the lifespan of the zebrafish used in our experiments may be accounted for by the increased cell apoptosis due to chronic inflammation, which was enhanced by the loss of *Sirt1*.

## Discussion

In this study, we investigated the function of *Sirt1* in zebrafish. We used the CRISPR/Cas9 system to knockout the *Sirt1* gene from the zebrafish genome. We designed a sgRNA targeting exon1 and found that the mutation was a 4-bp insertion and 20-bp deletion. We selected the 4-bp insertion line because maintenance of the 20-bp deletion line was cumbersome, but we are unable to explain why the maintenance of this line was difficult. The 4-bp insertion in exon1 led to a frame-shift mutation in *Sirt1* resulting in a truncated *Sirt1* protein that lacked the catalytic core domain consisting of two subdomains for NAD+ and substrate binding [20]. This *Sirt1* knockout was confirmed by using an antibody reactive to the zebrafish antigen. Therefore, we successfully established a *Sirt1*^-/-^ mutant zebrafish line.

An increase in ROS can directly or indirectly control SIRT1 activity [15,21]. Previous studies revealed a connection between *Sirt1* expression and ROS production in the regulatory scheme of age-related pathology [15]. Ralph et al. [22] showed that *Sirt1* overexpression protected heart tissue against oxidative stress. Hence, we expected the *Sirt1*^-/-^ mutant embryos to be weaker than wildtype embryos against oxidative stress and, thus, compared ROS sensitivity of wildtype and *Sirt1*^-/-^ mutant zebrafish embryos. Unexpectedly, after tBOOH treatment, wildtype embryos died earlier than *Sirt1*-knockout embryos. We measured H_2_DCFDA fluorescence and performed TUNEL staining for detection of ROS and apoptosis levels and found that ROS production was significantly increased in *Sirt1*^-/-^ embryos. Similarly, analysis of cell death using the TUNEL assay showed a significant mean effect on *Sirt1*^-/-^ embryos. Furthermore, when embryos were treated with ROS inhibitor NAC, we found NAC abated cell death induced by the lack of Sirt1. This might suggest that *Sirt1*^-/-^ mutant zebrafish show ROS resistance under abundant ROS production.

We performed qRT-PCT and western blotting to reveal the molecular changes in *Sirt1*^-/-^ mutant zebrafish. The experiments were performed on 4-dpf embryos to represent the embryonic stage and on 3-month old zebrafish to represent the adult stage. Three-months old zebrafish are considered fully mature. We investigated atrophic phenotype at 3-months of age and did not detect any obvious atrophy. The deterioration of the adult fish seemed to be caused by organ damages which is the result of repeated inflammation, cell damage and regeneration. Thus we carried out the experiment when the inflammation process is active and affected organs are not severely destroyed; therefore, we selected this age for analysis of representative mature adults.

Inflammation-related genes including IL-1b, IL-6 and TNF-α, were highly upregulated in *Sirt1*-knockout embryos. IL-1b, IL-6, NF-kB, TNF-α, and iNOS are known as aging-associated pro-inflammatory genes [23]. These genes are all upregulated through NF-kB activation on the aorta during aging [24]. Furthermore, by H&E staining of adult zebrafish, we found inflammatory cell infiltration occurring in the adjacent organs including the pancreas, intestine, and spleen. This was confirmed by MPO immunohistochemistry and TNF-α and NF-kB ISH results. Overall, our findings suggest that ROS induction caused by the lack of *Sirt1* led to chronic inflammation beginning early in embryo development. Additionally, we confirmed intestinal atrophy by H&E staining.

Similar to short lived *Sirt1*-knockout mice, recorded survival duration confirmed that Sirt1-knockout zebrafish showed a shorter lifespan than wildtype zebrafish. Furthermore, apoptotic events were confirmed using the TUNEL assay on 18-month-old zebrafish organ sections. We found a higher number of apoptotic cells in the *Sirt1*^-/-^ mutants than in the wildtype. Altogether, these results suggest that loss of *Sirt1* results in enhanced chronic inflammation and subsequent increased cell apoptosis, which together may account for the observed reduction in life span.

Overall, our data unequivocally revealed that *Sirt1*-lacking zebrafish were characterized by oxidative injury and chronic inflammation. The generation of ROS significantly increased in *Sirt1*^-/-^, causing inflammation followed by apoptosis and regeneration. As this cycle was repeated over and over, aging brought about atrophy; therefore, the *Sirt1*^-/-^mutation played a determinant role in the reduction of life span of zebrafish in this study. The pro-inflammatory function of *Sirt1* was accompanied by apoptosis and a shortened life span. Furthermore, we suggest that our mutant zebrafish model will be a useful tool in studies dealing with inflammatory effects.
